# Nutrition in Menopausal Women: A Narrative Review

**DOI:** 10.3390/nu13072149

**Published:** 2021-06-23

**Authors:** Thais R. Silva, Karen Oppermann, Fernando M. Reis, Poli Mara Spritzer

**Affiliations:** 1 Gynecological Endocrinology Unit, Division of Endocrinology, Hospital de Clínicas de Porto Alegre, Rua Ramiro Barcelos 2350, Porto Alegre 90035-003, Brazil; thaisrasia@gmail.com; 2 Laboratory of Molecular Endocrinology, Department of Physiology, Federal University of Rio Grande do Sul, Porto Alegre 90035-003, Brazil; 3 Medical School of Universidade de Passo Fundo, São Vicente de Paulo Hospital, Passo Fundo 99052-900, Brazil; karenoppermann@gmail.com; 4 Division of Human Reproduction, Hospital das Clínicas, Department of Obstetrics and Gynecology, Universidade Federal de Minas Gerais, Belo Horizonte 30130-100, Brazil

**Keywords:** menopause, nutrition, body composition, bone, cardiovascular risk

## Abstract

Among the various aspects of health promotion and lifestyle adaptation to the postmenopausal period, nutritional habits are essential because they concern all women, can be modified, and impact both longevity and quality of life. In this narrative review, we discuss the current evidence on the association between dietary patterns and clinical endpoints in postmenopausal women, such as body composition, bone mass, and risk markers for cardiovascular disease. Current evidence suggests that low-fat, plant-based diets are associated with beneficial effects on body composition, but further studies are needed to confirm these results in postmenopausal women. The Mediterranean diet pattern along with other healthy habits may help the primary prevention of bone, metabolic, and cardiovascular diseases in the postmenopausal period. It consists on the use of healthy foods that have anti-inflammatory and antioxidant properties, and is associated with a small but significant decrease in blood pressure, reduction of fat mass, and improvement in cholesterol levels. These effects remain to be evaluated over a longer period of time, with the assessment of hard outcomes such as bone fractures, diabetes, and coronary ischemia.

## 1. Introduction

Menopause is literally the ceasing of menstruation, but a broader definition includes “the permanent cessation of menstrual cycles following the loss of ovarian follicular activity” [1]. Climacteric is the transitional phase from the first signs of ovarian senescence until its complete installation. Among the various endocrine changes that characterize the progressive loss of ovarian function and ultimately lead to menopause, the most important is the decrease of circulating levels of ovarian steroids. The loss of luteal phase progesterone due to missed ovulation may cause menstrual irregularity and heavy menstrual bleeding in the late premenopausal years, while the subsequent decrease of estradiol levels due to follicular exhaustion is related to vasomotor symptoms, and the cause of urogenital atrophy, bone loss, and increased cardiovascular and metabolic risk [2,3]. Although menopause is a conspicuous event, the menopausal transition may span several years and the health impact of postmenopausal hypoestrogenism can extend for decades, even when symptoms are no longer present [4,5].

Menopause is associated with increased prevalence of obesity, metabolic syndrome, cardiovascular disease, and osteoporosis [3]. Weight gain is observed among midlife women and has been ascribed to both chronological aging and to the menopause transition [6]. Recent data from a large population-based cohort in the United States [7] reinforced the idea that weight gain is not only related to the menopause transition, even though the fat mass increases rapidly in this phase. In this sense, a population-based study that we conducted in southern Brazil showed that sedentariness rather than menopause is associated with a two-fold increased risk of overweight/obesity [8]. Therefore, exercise along with calorie restriction should be recommended in all those postmenopausal women with excess weight, for reductions in metabolic and cardiovascular risk [9].

The ability to switch from fat utilization during fasting to carbohydrate utilization during hyperinsulinemia is defined as metabolic flexibility [10]. Gonodal hormones might regulate metabolic flexibility at the level of the mitochondria, determining how nutrients are converted into energy [11]. In postmenopausal women, metabolic flexibility diminishes due to estrogen reduction and more fat accumulates in central depots [12].

The integral health care of menopausal women should therefore emphasize lifestyle assessment and counseling to counterbalance the negative effects of estrogen deficiency on general well-being and minimize the risk of metabolic syndrome, osteoporosis, bone fractures, and vascular events [2,3]. Among the various aspects of health promotion and lifestyle adaptation to the postmenopausal period, nutritional habits are essential because they concern all women, can be modified, and impact both longevity and quality of life. In this narrative review, we shall discuss the current evidence on the association between dietary patterns and clinical endpoints in postmenopausal women, such as body composition, bone mass, and risk markers for cardiovascular disease (CVD), including studies of risk association and/or effects of dietary interventions and thereby providing novel insight into the establishment of optimal dietary guidelines for healthy postmenopausal period.

In order to find relevant publications, a search was conducted in Pubmed with combinations of keywords and Medical Subject Headings (MeSH) “Diet”, “Recommended Dietary Allowances”, “Diet, Mediterranean”, “Diet, Fat-Restricted”, “Diet, Carbohydrate-Restricted”, “Glycemic Index”, “Body Composition”, “Menopause”, “Postmenopause”, and “Cardiovascular Diseases”. All articles published up to February 2021 were considered for eligibility.

## 2. Dietary Intake and Clinical Endpoints in Menopausal Women

### 2.1. Body Composition

In the menopausal transition, lowering estrogen levels have been associated with loss of lean body mass (LBM) and increase in fat mass (FM) [13,14]. In the longitudinal Study of Women’s Health Across the Nation, LBM loss during the menopausal transition averaged 0.5% (a mean annual absolute decrease of 0.2 kg), and FM increased by 1.7% per year (mean annual absolute increase of 0.45 kg) [7]. Body composition changes in this population were associated with increased risk of coronary heart disease, potentially compromising the woman’s health as a whole. In the National Health and Nutrition Examination Survey (NHANES), participants with low LBM and high FM had the highest cardiovascular and total mortality risk [15].

#### 2.1.1. Dietary Intake and Lean Body Mass in Postmenopausal Women

##### Dietary Protein

Ageing increases dietary protein requirements [16,17] because skeletal muscles reduce their capacity of activating protein synthesis in response to anabolic stimuli, possibly due to insulin resistance [18,19]. In fact, observational studies have indicated that higher protein intake is associated with higher LBM in postmenopausal women [20,21,22]. In the Women’s Health Initiative study, higher protein intake (1.2 g/kg body weight) was associated with a 32% lower risk of frailty and better physical function [23]. The mean protein intake associated with higher skeletal muscle mass index in postmenopausal women was 1.6 g/kg body weight [22], although the Institute of Medicine recommends for all ages the protein allowance of 0.8 g/kg body weight [24]. Because observational results are unable to determine the direction of cause and effect, randomized controlled trials (RCT) have been developed to validate this hypothesis. A meta-analysis of 36 RCTs with 1682 participants concluded that protein supplementation, from 6 to 78 weeks, does not lead to increase in LBM in non-frail community-dwelling older adults [25]. The few available interventional studies focusing on postmenopausal women have shown that high protein intake did not promote LBM gain when compared to recommended dietary allowance (RDA) (Table 1) [26,27]. Indeed, beyond the metabolic and physiological changes of aging that may alter protein metabolism [28], the current evidence suggests that RDA may be sufficient to maintain LBM in older women.

While LBM maintenance cannot be attributed to high dietary protein intake in healthy postmenopausal women, it could be associated, at least in part, with healthy dietary patterns, such as the Mediterranean diet (MD).

##### Mediterranean Dietary Pattern

Through acting directly in oxidative stress [29], inflammation [30,31], and insulin resistance [18,19], regarded as risk factors for muscle catabolism, the MD components have been associated with better muscle measurements in postmenopausal women [32,33,34].

**Figure 1:**
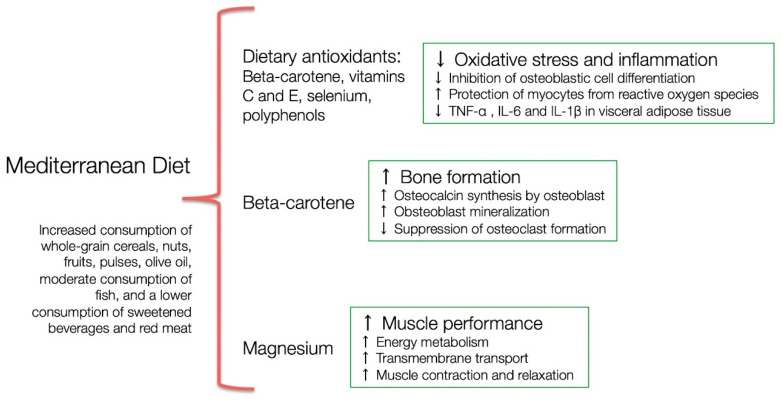
Potential benefits of Mediterranean diet on body composition in postmenopausal women. Redrawn and modified from [34].

In a recent review, Granic et al. [35] hypothesized that the ‘myoprotective’ effect of the MD could be linked to higher intake of plant-based foods because they combine nutrients that act together to preserve the muscles. In a previous work, we have also proposed a model for the potential benefits of MD on body composition in postmenopausal women. The presence of antioxidants like beta-carotene, as well as vitamins C and E protects from deleterious effects of oxidative stress, while magnesium improves energy metabolism, transmembrane transport, and skeletal muscle function [34] (Figure 1).

However, studies about MD intervention focusing on LBM gain or maintenance in postmenopausal women were not available until now, expressing an important gap regarding this issue. Therefore, further research is needed on the potential effects of non-protein nutrients on muscle health in older women. 

#### 2.1.2. Dietary Intake and Fat Mass in Postmenopausal Women

##### Dietary Carbohydrate, Whole Grains, and Glycemic Index

The role of dietary carbohydrate for promoting FM loss remains to be elucidated. In obese individuals, a previous systematic review has shown that mild low carbohydrate diet (40% of total energy) was not associated with decrease in fat mass [36]. Recently, a randomized control trial with 57 women (age 40 ± 3.5 years, BMI 31.1 ± 2.6 kg∙m^−2^) yielded similar results, with low-carbohydrate-high-fat diet having no superior effect on FM in comparison to a normal diet [37]. However, some carbohydrate sources can be beneficial, while others are not, depending at least in part on their fiber content [38]. In an RCT with 81 men and 32 postmenopausal women, the consumption of whole grains during six weeks had positive effects on the resting metabolic rate and stool energy excretion, which influenced favorably the energy balance [39]. Indeed, this study adds support for dietary guidance recommending the consumption of whole grains instead of refined grains in order to reduce adiposity [40], although there are very few interventional studies focusing on postmenopausal women.

Complementing additional ways of characterizing carbohydrate foods, such as fiber and whole grain content, glycemic index (GI) should also be considered particularly important in reducing total body FM and managing weight [38]. Eating a meal with high GI elicits a quick pancreatic response to the rising blood glucose levels, with intense insulin secretion that rapidly lowers blood glucose and causes hunger and overeating [41]. In fact, a Cochrane systematic review including data from 202 overweight or obese men and women in six RCTs reported a significantly greater decrease in total FM in the low GI diet than in control diet groups [42]. Specifically in postmenopausal women, a clinical trial with low GI (<55) dietary intervention, aimed to balance energy needs, has shown that, despite similar energy intake and resting metabolic rates during the six months of follow-up, all participants lost total body and regional FM [43].

##### Mediterranean Dietary Pattern

An umbrella review of meta-analyses reported evidence suggesting greater effectiveness of MD in reducing body weight and waist circumference when compared to control diets [44]. However, the evidence regarding the MD effect on FM was scarce. In a cross-sectional study with 176 perimenopausal women from the FLAMENCO project, a higher MD adherence, an increased consumption of whole-grain cereals, nuts, fruits, pulses, whole dairy products, and olive oil, and a lower consumption of sweetened beverages were associated with lower FM [45] (Figure 1). In a non-controlled clinical trial, 89 women (46 in reproductive age and 43 postmenopausal) were prescribed hypocaloric traditional MD for eight weeks and obtained an average reduction of 2.3 kg in FM, suggesting that postmenopausal women can lose FM with this diet in the same way as younger women [46]. However, the potential role of MD in reducing FM in comparison to other dietary patterns needs to be further evaluated.

In contrast, The Women’s Health Initiative Dietary Modification trial have found that a low-fat (≤20% of total energy) diet was related with greater reductions in percentage body fat and FM after one and three years of follow-up [47]. Indeed, trials where participants, men and women, were randomized to a lower fat intake (≤30% of total energy) showed a consistent, stable but small effect on percentage body fat compared with higher fat arms, as published in a Cochrane systematic review [48]. Despite MD being associated with higher dietary fat intake, both MD and low-fat diet are often associated with increased intake of vegetables, fruits and grains. Recently, a crossover RCT showed that a plant-based, low-fat diet promoted greater decrease in FM than an animal-based, ketogenic diet [49]. However, the study enrolled only 20 adults and the primary outcome was daily ad libitum energy intake between each two-week diet period. In summary, low-fat, plant-based diets are associated with beneficial effects on FM, and future studies are needed to confirm these results in postmenopausal women.

### 2.2. Bone Health

The decrease in bone mineral density (BMD) that accompanies aging is related to declining reproductive hormone concentrations [50,51]. BMD loss accelerates markedly along the late perimenopause, when menses become more irregular [52].

Several studies have shown the importance of adequate calcium and vitamin D intake for better BMD and prevention of osteoporosis and fractures in older adults [53]. However, the recommended daily intake of calcium for older adults ranges from 700 mg in the UK [54] to 1200 mg in the US [55], and the North American Menopause Society actually recommends 1000 to 1500 mg of dietary calcium per day to postmenopausal women [56]. Available evidence from completed RCTs provided no support for the use of vitamin D or calcium supplementations alone to prevent fractures. On the other hand, daily supplementation with both vitamin D (400–800 IU/day) and calcium (1000–1200 mg/day) was a more promising strategy [57].

Besides, analysis of isolated nutrients is not sufficient to reveal the complex interactions between nutrients and non-nutrients contained in food. Therefore, the study of dietary patterns, particularly the MD pattern, has been proposed to investigate the relationship between diet and BMD.

Previous studies showed that better adherence to the MD is positively associated with BMD in middle-aged and elderly people [58] and in postmenopausal women [34,59]. Recent findings from an RCT undertaken across five European centers support these results from observational studies. In this trial, a MD-like diet prescribed for one year and accompanied by individual advice and supplies of the required foods produced a significant decrease in the rate of BMD loss among people with osteoporosis, compared to a group that received only informative leaflets [60].

The potential benefits of the MD for BMD may result from the combined presence of nutrients and non-nutrients components. Dietary intake of carotenoids has been associated with BMD [61]. Indeed, beta-carotene seems to suppress osteoclast formation and bone resorption [62]. Vitamin K also plays a role in bone formation through osteocalcin synthesis by osteoblasts, which is a vitamin K dependent protein [63]. However, concerns have been raised about the integrity of some vitamin K supplementation studies [64]. In addition, a recent RCT of vitamin K (MK-7) or placebo supplementation in postmenopausal women observed no difference in bone turnover markers and microstructure between the groups during three years of follow-up [65]. Regarding vitamin C, a recent meta-analysis of observational studies reported that greater dietary vitamin C intake was associated with a lower risk of hip fracture and osteoporosis, as well as higher BMD at femoral neck and lumbar spine [66]. Moreover, a review of Mendelian randomization-based studies examined potential associations between serum nutritional factors and BMD. Higher selenium levels positively influence BMD at specific skeletal sites, suggesting that selenium plays a crucial role in bone metabolism [67]. Therefore, an adequate consumption of beta-carotene, vitamin C, and selenium trough MD could lead to better BMD (Figure 1). In contrast, processed food pattern (high intakes of meat pies, hamburgers, beer, sweets, fruit juice, processed meats, snacks, spirits, pizza and low intake of cruciferous vegetables) was inversely associated with bone mineral content in a cohort study with 347 women (aged 36–57 years) [68].

In a nutshell, the data above suggest that a MD pattern, combined with other healthy lifestyle habits, may be a useful non-pharmacological strategy for the primary prevention of osteoporosis and fractures in the postmenopausal period.

### 2.3. Cardiovascular Risk

The estrogens secreted by the ovaries during the reproductive period exert protective effects on vascular endothelial function as well as on lipid metabolism. After menopause, the relative estrogen deprivation contributes to increase vascular tone through both endocrine and autonomic mechanisms that converge impaired nitric oxide dependent vasodilation [1,69].

While CVD risk increases with menopause, this is difficult to distinguish from the effect of ageing [70]. Nonetheless, the use of menopausal hormone therapy (MHT) has been associated with protective effect against coronary artery calcification [71] and slower progression of carotid artery intima-media thickness, both of which are markers of subclinical CVD [72].

**Figure 2:**
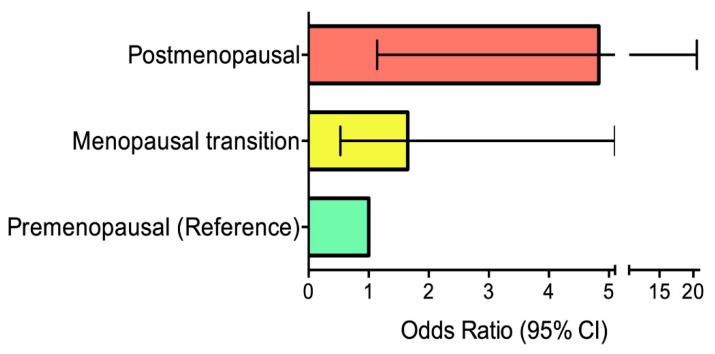
Adjusted odds ratios (with 95% confidence intervals) for central adiposity, defined as waist circumference ≥ 88 cm, in women in the menopausal transition and postmenopausal women. Adapted from [13].

Postmenopausal women have two to three times higher prevalence of metabolic syndrome, compared to similar aged premenopausal women [73]. The changes on cardiovascular risk begin during the perimenopause period. Menopause transition results in lipid profile changes, with a 10–15% higher LDL-cholesterol and triglyceride levels and slightly lower HDL cholesterol levels [74]. This period also accounts for an increase in BMI and abdominal adiposity, with postmenopausal women presenting approximately five times the risk of central obesity compared to premenopausal women [13] (Figure 2). The presence of central obesity has been associated with decreased heart rate variability, another marker of subclinical CVD [75].

Furthermore, there is an increase in blood pressure after menopause that may be a direct effect of hormonal changes on the vasculature and metabolic changes with ageing [69]. Sodium sensitivity increases during menopausal transition, frequently leading to intermittent fluid retention (edema of the legs, hands, and lower eyelids), contributing to higher cardiovascular risks [76].

Diet is a major modifiable risk factor for CVD. The traditional approach of nutritional epidemiology focuses on the potential impacts of individual foods or nutrients. Scientific societies recommend the following healthy dietary pattern to decrease the risk of major chronic diseases and increase overall wellbeing (Table 2): protein sources primarily from plants, nuts, fish, or alternative sources of omega-3 fatty acids; fat mostly from unsaturated plant sources; carbohydrates primarily from whole grains; at least five servings of fruits and vegetables per day; and moderate dairy consumption as an option [77].

The American Heart Association (AHA) suggests the following dietary targets to improve cardiovascular health: fruits ≥ 4.5 cups/day, fish and shellfish ≥ 200 g/week, sodium ≤ 1500 mg/day, sugar-sweetened beverages ≤ 36 fl oz/week, whole grains 3 or more 1-oz-equivalent servings/day, nuts, seeds, and legumes ≥ 4 servings/week (Table 2) [78,79,80]. In a recent meta-analysis of cohort studies, higher intakes of fruit and vegetables were associated with lower mortality rates, supporting current dietary recommendations to increase intake of fruits and vegetables, but not fruit juices and potatoes [81].

Although diet could be a powerful intervention to reduce cardiovascular risks in postmenopausal women, the studies could not clearly demonstrate this action on the arteries. The Study of Women’s Health Across the Nation [82] evaluated the prospective associations between empirically derived dietary patterns during midlife and subclinical carotid atherosclerosis later in life among women. After extensively adjusting for covariates, higher adherences to Western dietary patterns (e.g., rich in dairy products, pizza, read meat, and salad dressing and poor in fruits, skimmed milk, legumes, cruciferous vegetables, and tomatoes) were associated with increased common carotid artery intima-media thickness (CCA-IMT). Prudent diet (e.g., including dark yellow vegetables, green leafy vegetables, cruciferous vegetables, legumes, and fruits and avoiding whole milk, margarine, organ meats, sweets, and beer) was not associated with CCA-IMT. The adoption of a diet low in red meat, processed meat, deep-fried products, and sugar-sweetened beverages among midlife women is associated with a lower future risk of atherosclerosis.

Low-energy diet is also recommended for postmenopausal women to prevent metabolic alterations [83]. In a cross-sectional study of 4984 women aged 30–79 years, three dietary patterns (Western, healthy, and traditional) were identified. In a stratified analysis by menopausal status, the inverse association of the healthy dietary pattern (characterized by high factor loadings with green-yellow vegetables, healthy-protein foods, seaweeds, and bonefish) and metabolic syndrome was statistically significant only among postmenopausal women. In assessing each component of metabolic syndrome, the healthy dietary pattern was found to be protective for blood pressure and triglyceride levels among premenopausal women and for obesity and HDL-cholesterol levels among postmenopausal women [84].

A reduction in energy expenditure during midlife can also cause obesity during menopause [83]. According to a four-year follow-up study, the decrease in physical activity began two years before menopause. Aging resulted in gained subcutaneous abdominal fat over time to all women, however, only those who became postmenopausal had a significant increase in visceral abdominal fat [13,85].

In a population-based cross-sectional study involving 292 Brazilian women, we have shown a higher risk of overweight/obesity for inactive women. Sedentariness increased the risk of diabetes mellitus and metabolic syndrome after adjustment for menopausal status and other potential confounders [8]. In addition, active postmenopausal women seem to have healthier dietary choices than their sedentary counterparts, such as foods with higher intake of protein and lower intake of chips and refined grains [86].

During the menopause transition there is a tendency to weight gain accompanied by an increase in central fat distribution that continues into the post-menopause [13,87]. For postmenopausal women, sedentary lifestyle and a diet with carbohydrate intake accounting for more than 55% of total energy contribute to higher cardiovascular risk, according to high sensitivity C-reactive protein levels [88].

The best diet for weight loss is still debatable, whether low-fat, low-carbohydrate, or high-protein diet, with no evident superiority of one over the others for the specific purpose of losing weight [87]. The obesity-management guidelines from the American College of Cardiology/American Heart Association Task Force on Practice Guidelines and the Obesity Society recommend a daily caloric deficit of 500 to 750 kcal, which for most women means eating 1200 to 1500 kcal/d, and is expected to result in an average weight loss 0.5 to 0.75 kg/wk [89].

Although the differences on cardiometabolic risk are small, the macronutrient composition of the weight loss diet may affect some intermediate outcomes. Low-fat diets tend to reduce low-density lipoprotein (LDL) cholesterol levels, while low-carbohydrate diets may be more effective to low triglycerides and increase high-density lipoprotein cholesterol levels [87].

Nevertheless, MD has the advantage of combining weight loss with CVD risk reduction [90]. This diet reduces the consumption of saturated animal fats in favor of unsaturated vegetable fats and a high intake of polyphenols and n-3 fatty acids with anti-inflammatory and antioxidant properties [91]. The phenolic compounds (polyphenols) are presented in extra virgin olive oil, whole grain cereals, nuts, legumes, vegetables, red wine, and fruits. Due to their antioxidant and anti-inflammatory properties, the synergistic consumption of these Mediterranean foods could represent an ideal nutritional pattern in menopause [92]. Evidence from observational studies and randomized trials consistently shows a small but significant decrease in LDL cholesterol as well as systolic and diastolic blood pressure in association with the MD. This diet has also been linked to reduced risk of CVD (including coronary disease) and CVD death among different female cohorts, although more evidence is required for these outcomes in postmenopausal women [93].

Concerning weight loss, the effect of hypocaloric MD is compared with a low-fat diet, a low-carbohydrate diet and the American Diabetes Association diet [90]. On top of that, peri- and postmenopausal women showed a high adherence to MD [92], which could increase the results on weight and cardio-metabolic profile.

In addition, we recently reported higher isoflavone dietary intake may be associated with lower risk of subclinical CVD, as assessed by CCA-IMT status, independently of endogenous estradiol levels and BMI [94]. Isoflavones might have beneficial effects by its anti-inflammatory and antioxidant properties, through the production of equol, an active metabolite formed from daidzin/daidzein by gut microbiota [95].

In general, the changes in weight and fat distribution in women are associated with aging and mainly with the decrease in estradiol levels during peri- and post-menopause. There are also changes in physical activity contributing to the accumulation of weight and body fat. At the same time, changes in cholesterol and triglyceride levels also favor increased cardiovascular risk.

In summary, physical activity and diet are modifiable factors in the quest for cardiovascular protection. Weight loss diets in overweight or obese women improve the metabolic syndrome in its various parameters. MD is composed of healthy foods that have anti-inflammatory and antioxidant properties. In addition to these benefits, it seems to incorporate greater adherence of women over time. These effects remain to be evaluated over a longer period of time, with the study of hard outcomes such as coronary ischemia.

## 3. Summary and Research Perspectives

- Menopausal transition has been associated with loss of BMD, LBM and increase of FM;
- RDA for protein intake may be sufficient to maintain LBM; Mediterranean diet components could be linked with better LBM;
- Low-carbohydrate-high-fat diet should not be recommended in order to reduce FM;
- In overweight or obese women, low GI diet could lead to greater decrease in FM than control diets;
- Future studies evaluating the effects of low-fat, plant-based diets on FM in postmenopausal women are needed;
- Mediterranean diet might significantly reduce the rate of BMD loss in women with osteoporosis;
- Calcium, vitamin D, vitamin K, selenium, magnesium, and beta-carotene adequate intake could be linked with better BMD in postmenopausal women;
- Diet is a major modifiable risk factor for CVD and could be a powerful intervention to reduce cardiovascular risks in postmenopausal women;
- Low-energy diet is recommended for postmenopausal women to prevent metabolic disturbance;
- Low-fat diets may lead to greater improvement in LDL cholesterol levels, whereas low-carbohydrate diets may result in greater improvement in triglyceride and HDL cholesterol levels;
- Mediterranean diet is associated with a small but significant decrease in blood pressure and reduced CVD risk of among different female cohorts, although more evidence is required for these outcomes in postmenopausal women.

## Figures and Tables

**Table 1:** Randomized controlled trials about the effect of high protein diets on LBM in postmenopausal women.

Author/Year	Country	Arms/Comparators	Duration	Participants	Interventions	LBM Analyses
Iglay, 2009	USA	HP: 1.2 g/kg body weight	12 weeks	36 postmenopausal women and men	HP diet + resistance training vs.	LBM increased: 1.1 ± 0.2 kg
		NP: 0.9 g/kg body weight		age = 61 ± 1 years	NP diet + resistance training	no difference between the groups
Rossato, 2017	Brazil	HP: 1.2 g/kg body weight	10 weeks	23 postmenopausal women	HP diet + resistance training vs.	HP LBM: 37.1 ± 6.2 to 38.4 ± 6.5 kg
		NP: 0.8 g/kg body weight		age = 63.2 ± 7.8 years	NP diet + resistance training	NP LBM: 37.6 ± 6.2 to 38.8 ± 6.4 kg
						no difference between the groups (*p =* 0.572)
Silva, 2020	Brazil	HP: 1.6 g/kg body weight	6 months	26 postmenopausal women	HP diet vs. NP diet	HP LBM: 35.6 ± 0.7 to 35.7 ± 0.7 kg
		NP: 0.8 g/kg body weight		age = 70.8 ± 3.6 years		NP LBM: 35.3 ± 0.7 to 35.4 ± 0.7 kg
						no difference between the groups (*p =* 0.683)

LBM: lean body mass; HP: high protein diet; NP: normal protein diet.

**Table 2:** Healthy diet recommendations.

Guideline	Proteins		Fats		Carbohydrates	
	Yes	No or Moderate	Yes	No	Yes	No
EAT Lancet Commission [77]	Protein from plants Legumes Nuts Fish Fruits	Red meat Processed meat Poultry and eggs Dairy products	Fat mostly from unsaturated plant sources	Saturated fats Partly hydrogenated oils	Carbohydrates primarily from whole grains	Refined grains Sugar
American Heart Association [78]	Fish ≥ 200 g/week	Processed meats ≤ 100 g/week	Nuts, seeds, and legumes ≥ 4 servings/week	Saturated fat ≤ 7% energy	Whole grains ≥ 3 servings/day Fruits ≥ 4.5 cups/day	Sodium ≤ 1500 mg/d Sugar-sweetened beverages ≤ 36 fl oz/ week

## Data Availability

Not applicable.
